# Human plasma-derived C1 esterase inhibitor concentrate has limited effect on house dust mite-induced allergic lung inflammation in mice

**DOI:** 10.1371/journal.pone.0186652

**Published:** 2017-10-16

**Authors:** Ingrid Stroo, Jack Yang, Adam A. Anas, J. Daan de Boer, Gerard van Mierlo, Dorina Roem, Diana Wouters, Ruchira Engel, Joris J. T. H. Roelofs, Cornelis van ‘t Veer, Tom van der Poll, Sacha Zeerleder

**Affiliations:** 1 Department of Immunopathology, Sanquin Research, Amsterdam, the Netherlands; 2 Center for Experimental and Molecular Medicine, Academic Medical Center, University of Amsterdam, Amsterdam, the Netherlands; 3 Department of Pathology, Academic Medical Center, University of Amsterdam, Amsterdam, the Netherlands; 4 Division of Infectious Diseases, Academic Medical Center, University of Amsterdam, Amsterdam, the Netherlands; 5 Department of Hematology, Academic Medical Center, University of Amsterdam, Amsterdam, the Netherlands; Centre National de la Recherche Scientifique, FRANCE

## Abstract

C1 esterase inhibitor (C1-INH) can inhibit multiple pathways (complement, contact-kinin, coagulation, and fibrinolysis) that are all implicated in the pathophysiology of asthma. We explored the effect of human plasma-derived C1-INH on allergic lung inflammation in a house dust mite (HDM) induced asthma mouse model by daily administration of C1-INH (15 U) during the challenge phase. NaCl and HDM exposed mice had comparable plasma C1-INH levels, while bronchoalveolar lavage fluid (BALF) levels were increased in HDM exposed mice coinciding with slightly reduced activation of complement (C5a). C1-INH treatment reduced Th_2_ response and enhanced HDM-specific IgG_1_. Influx of eosinophils in BALF or lung, pulmonary damage, mucus production, procoagulant response or plasma leakage in BALF was similar in both groups. In conclusion, C1-INH dampens Th2 responses during HDM induced allergic lung inflammation.

## Introduction

Asthma is a chronic airway inflammatory disease that results from an excessive immune response to common environmental allergens such as house dust mite (HDM)[1]. In 2013, the WHO estimated that worldwide 235 million people suffer from asthma and the incidence is still rising[2]. Symptoms include recurrent episodes of wheezing, coughing, chest tightness and shortness of breath in response to an allergen. Currently, asthma cannot be cured, however, a combination of inhaled corticosteroids (to suppress inflammation) and a short- or long-acting β-adrenergic agonist (to open the constricting bronchial smooth muscle cells) can control the disease and improve quality of life[3]. While the majority of patients with asthma can be treated effectively with the currently available medications, adequate disease control cannot be achieved in a significant proportion of patients. Because of the high incidence and burden on our health care system, there is an urgent need to explore new treatment options.

The complement system is one of the inflammatory pathways activated during asthma. The complement cascade consists of a number of plasma- and membrane-bound proteins that can be activated via three distinct routes; the classical, lectin or alternative pathway of complement. All three pathways converge at the level of C3 which following activation subsequently activates C5. This activation cascade leads to the formation of the anaphylatoxins C3a and C5a. Anaphylatoxins possess many proinflammatory and immunomodulatory characteristics; for example they are chemotactic factors for eosinophils[4]. In the bronchoalveolar lavage fluid (BALF) of asthmatic patients C3a and C5a levels are increased following allergen challenge and both anaphylatoxins correlate with influx of eosinophils[5]. Moreover, experimental asthma models suggest that C3a and C5a regulate Th_2_ response during the sensitization and challenge phase[6].

Activation of both the classical and lectin pathway of complement is tightly regulated by C1 esterase inhibitor (C1-INH). The plasma glycoprotein C1-INH belongs to the family of serpins (serine protease inhibitors). Serpins are characterized by a typical mechanism of action; target proteases attack the “fake” substrate conformation of the serpin, leading to the formation of a covalent complex between protease and serpin (deadly handshake)[7]. Next to complement inhibition, C1-INH is 1) a major regulator of the contact-kinin system by blocking of activated factor XII (FXIIa) and plasma kallikrein, 2) the main inhibitor of activated factor XI (FXIa), the central player in the intrinsic coagulation, and 3) an inhibitor of fibrinolysis via blocking plasmin and tissue-type plasmin activator[7]. These functions of C1-INH are all exerted via its protease inhibitor domain. However, several experimental studies have shown an inhibitory effect for C1-INH in the production of cytokines and attraction of leukocytes that is independent of its protease inhibitory activity[8–10].

Asthma is associated with activation of the coagulation system[11] and contact-kinin system[12]. The contact-kinin system is an interesting target for the treatment of asthmatic exacerbations. Activation of this system leads to the formation of bradykinin, a small molecule that causes smooth muscle contraction, increases vascular permeability, and enhances mucus secretion[12]. In a sheep model of allergen-induced airway inflammation, the bradykinin B_2_-receptor antagonist NPC349 blocked the airway hyperresponsiveness and reduced inflammatory mediators [13, 14]. Recently, a bradykinin B_1_-receptor antagonist impaired eosinophil influx in a murine ovalbumin asthma model[15]. Although these results are promising, studies exploring the role of the contact-kinin system in asthma are limited.

Taken together, as C1-INH targets multiple biological systems that are activated during asthma (being complement, contact-kinin, and coagulation pathways) it might be a promising therapy to alleviate asthmatic symptoms. Moreover, C1-INH is already used by patients suffering from hereditary angioedema and is proven to be safe and efficacious in humans. In the present study we treated mice that were subjected to our HDM asthma model during the challenge phase daily with human plasma-derived C1-INH and determined the inflammatory response.

## Materials and methods

### Mice

Female C57Bl/6J wild-type (WT) mice were purchased from Charles River Inc. (Maastricht, The Netherlands). Mice were housed under specific pathogen-free conditions receiving food and water *ad libitum*. Age-matched mice were used in all experiments. The Animal Care and Use Committee of the University of Amsterdam approved all experiments (permit numbers: DIX102020AQ and DIX102791). Intranasal inoculation was performed under isoflurane anesthesia and mice were euthanized under general anesthesia, all efforts were made to minimize suffering.

### Kinetics C1-INH

Nine-weeks old mice received a single injection of 5 U (1 U equals ~250 μg) or 15 U of human plasma-derived C1-INH (Cetor, Sanquin, Amsterdam, Netherlands) intravenously (i.v.) or intraperitoneally (i.p.). Mice were randomly assigned to 3 groups and blood samples were taken at 1 and 2 hours (group I), 6 and 10 hours (group II), or 48 hours (group III) from the facial vein into EDTA tubes (Microvette®, Sarstedt, Etten-Leur, Netherlands) (n = 3 per group). Mice were euthanized and blood was obtained from the vena cava inferior 4 (group I), 24 (group II) or 72 hours (group III) following injection. Blood samples were centrifuged to collect plasma. All samples were stored at -80°C until further analysis.

### HDM asthma model

HDM allergen whole body extract (Greer Laboratories, Lenoir, N.C., USA), derived from the common European HDM species *Dermatophagoides pteronyssinus*, Der p, was used to induce allergic lung inflammation as described previously[16]. In short, 8-weeks old mice (n = 10) were inoculated intranasally on day 0, 1 and 2 with 25 μg HDM (sensitization phase) and on day 14, 15, 18 and 19 with 6.25 μg HDM (challenge phase). Controls (n = 6) received sterile saline intranasally on each occasion. Inoculum volume was 20 μl for every HDM and saline exposure and inoculation procedures were performed under isoflurane inhalation anesthesia. During the challenge phase (day 14 till 19) mice received daily 15 U human plasma-derived C1-INH i.p. or vehicle (provided by Sanquin) containing all excipients of C1-INH (i.e. sodium chloride, saccharose, sodium citrate, L-Valine, L-Threonine, L-Alanine). Both NaCl and HDM exposed mice were treated at the same days; all mice were included in a single experiment. Mice were euthanized at day 20 and blood was collected from the vena cava inferior into citrate (4:1 v/v), after which 10 mM EDTA, 10 mM benzamidine, and 0.2 mg/ml SBTI (all end concentrations) was added to specifically inhibit coagulation, contact, and complement activation. One-sided BALF was collected by instilling and retrieving 0.8 ml of sterile PBS containing 10 mM EDTA, 10 mM benzamidine and 0.2 mg/ml SBTI in 400 μl aliquots via the trachea. Cell counts were determined for each BALF sample in a hemocytometer (Beckman Coulter, Fullerton, CA, USA) and differential cells counts by cytospin preparations stained with Giemsa stain (Diff-Quick; Dade Behring AG, Düdingen, Switzerland). BALF supernatant was collected for protein analysis. The right lung was fixed 24 hours in 10% formalin. The mediastinal lymph nodes (MLN) were dissected and single cell suspension were prepared by mashing the cells through a 70 μm cell strainer. Cell counts were determined in a hemocytometer for each MLN sample and 2x10^5^ cells/well were seeded in a 96-well round bottom plate and restimulated for 4 days with 50 μg/ml HDM or NaCl. Supernatants were collected for cytokine production.

### Histology

Formalin-fixed tissue was embedded in paraffin using standard procedures. Four μm thick sections were cut and used for all (immuno)histochemical stainings. For examining allergic lung inflammation, sections were stained with Hematoxylin and Eosin (HE) and analyzed by a pathologist in a blinded fashion. HE-stained sections were scored for interstitial inflammation, peribronchial inflammation, perivascular inflammation, and edema on a scale from 0–4 (0: absent; 1: mild; 2: moderate; 3: severe; 4: very severe)[16]. Total pathology score was expressed as the sum of the scores for the different parameters. For examining mucus production, sections were stained with periodic acid-Schiff reagens after diastase digestion (PasD). The amount of mucus per lung section was assessed, by a pathologist in a blinded fashion, semi-quantitatively on a scale from 0–8 (0–4 for mucus plug formation; 0–4 for the extent of goblet cell hyperplasia)[16]. For eosinophil staining, sections were digested for 20 min in 0.25% pepsin in 0.1 M HCl, incubated overnight at 4°C with rabbit-anti-mouse MBP (Major Basic Protein; kindly provided by Dr. Nancy Lee and Prof. James Lee, Mayo Clinic Arizona, Scottsdale, Ariz., USA)[17]. Next, sections were incubated for 30 min with poly HRP-anti-rabbit IgG (Brightvision, Immunologic, Duiven, the Netherlands) and stained using 3,3”-diaminobenzidine dihydrochloride (DAB). Entire sections were digitized with a slide scanner using the 10x objective (Olympus dotSlide, Tokyo, Japan). Influx of eosinophils was determined by measuring the MBP positive area by digital image analysis (ImageJ 1.43, National Institute of Health, Bethesda, MD, USA; http://rsb.info.nih.gov/ij/) and expressed as a percentage of the total lung area.

### Assays

Human C1-INH antigen (Ag) and activity (Act) were measured in plasma by ELISA using as capture antibody monoclonal mouse anti-human C1-INH (clone RII, Sanquin) and as detection antibody biotinylated rabbit anti-human C1-INH (Sanquin) or biotinylated C1s (Calbiochem, Merck Millipore, Amsterdam, Netherlands) respectively[18]. Normal human plasma with known C1-INH concentration was used as standard. Complement activation in plasma and BALF was determined by C5a ELISA. Purified rat anti-mouse C5a (clone I53-1486) was used as capture antibody, biotinylated rat anti-mouse C5a (clone I52-278) was used as detection antibody (all from BD Biosciences). A standard curve for C5a was generated by serial dilutions of an in-house standard of maximal activated mouse serum, by incubating normal mouse serum at 37°C for 1 week in the presence of sodium azide. Purified recombinant mouse C5a (BD Biosciences) was used to determine concentration of C5a in maximal activated mouse serum. Plasma total IgE was determined using rat-anti-mouse IgE as capture antibody, purified mouse IgE as standard and biotinylated rat-anti-mouse IgE as detection (all from BD Biosciences, Pharmingen, Breda, the Netherlands). Plasma HDM specific IgG_1_ was determined using HDM as capture, and biotinylated rat-anti-mouse IgG_1_ (from BD Biosciences) as detection. HDM specific IgG_1_ is expressed as percentage compared to WT HDM group. IL-4, IL-5, IL-13 (MLN supernatants) and E-selectin were measured using DuoSet ELISA kits (R&D Systems, Abingdon, UK) according to the supplied protocol. BALF IL-4, IL-5 and IL-13 were determined by a Mouse Magnetic Luminex Screening Assay according to the manufacturer’s protocol (R&D Systems) and analyzed on a Bio-Rad BioPlex® 200 (Bio-Rad Laboratories, Veenendaal, the Netherlands). BALF IgM was determined using rat-anti-mouse IgM as capture antibody, purified mouse IgM as standard and biotinylated goat-anti-mouse IgM as detection (all from BD Biosciences). BALF total protein was determined using Bio-Rad Protein Assay (Bio-Rad Laboratories). Thrombin anti-thrombin complexes (TATc) were measured by ELISA according to manufacturer’s instructions (Stago BNL, Leiden, the Netherlands).

### Statistical analysis

Results are expressed as mean ± standard error of the mean (SEM). Comparison between two groups was done by Mann-Whitney U test. For experiments with more than two groups, the Kruskal-Wallis test was used as a pretest, followed by Mann-Whitney U test where appropriate. All statistical analyses were performed using GraphPad Prism 6 (GraphPad Software, San Diego, CA, USA). Values of *P* ≤ 0.05 were considered statistically significant.

## Results

### C1-INH plasma levels after single injection

**Fig 1:**
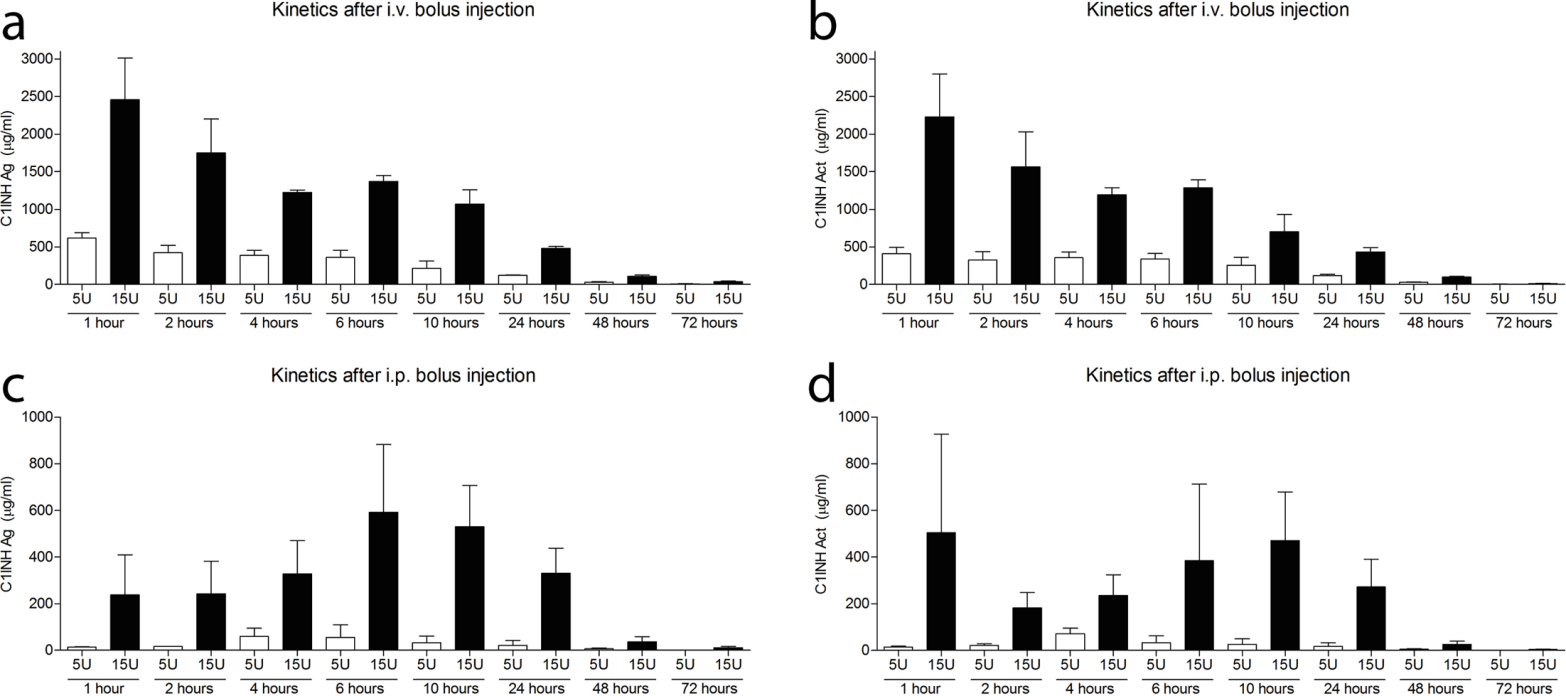
C1-INH plasma levels after i.v. or i.p. bolus injection. Human plasma derived C1-INH antigen (Ag) and activity (Act) in plasma after bolus injection of 5U (white bars) or 15U (black bars) C1-INH either i.v. (A-B) or i.p. (C-D) at indicated time points up to 72 hours following injection. Data are presented as mean ± SEM, n = 3 per time point.

In order to design a C1-INH treatment schedule for our mouse asthma model, we needed to obtain more information about the behavior of this human plasma derived protein in mice. Therefore mice were injected with a single dose of C1-INH (5 U or 15 U) either i.v. or i.p. At indicated time points up to 72 hours after injection, blood was collected and C1-INH antigen and activity levels were determined in plasma (Fig 1A–1D). Following i.v. injection, C1-INH antigen levels declined gradually with an estimated elimination half-life of 10 hours for both doses. Plasma levels of C1-INH antigen was on average 4.0 ± 0.2 fold higher in 15 U compared with 5 U treated mice. After i.p. administration of C1-INH, plasma antigen levels increased steadily reaching after approximately 6 hours the maximal plasma concentration (55 μg/ml for 5 U, and 600 μg/ml for 15 U). Plasma levels of C1-INH antigen following i.p. administration were on average 12.8 ± 1.7 fold higher in 15 U compared with 5 U dosage. Elimination half-life after i.p. administration was approximately 12 hours for both doses. C1-INH activity was comparable with antigen level for both routes and doses, indicating that C1-INH was still biologically active.

For our experimental HDM asthma mouse model, our goal was to achieve plasma levels of 500–1000 μg/ml (comparable to 2- to 4-times normal human plasma levels, i.e. ~250 μg/ml). Additionally, we were aiming for stable plasma levels throughout the whole experiment, which is more easily reached with i.p. injections. To fulfill these criteria, a treatment schedule of daily i.p. injections with 15 U C1-INH was chosen.

### Daily C1-INH treatment results in local C1-INH that slightly inhibits complement activation

**Fig 2:**
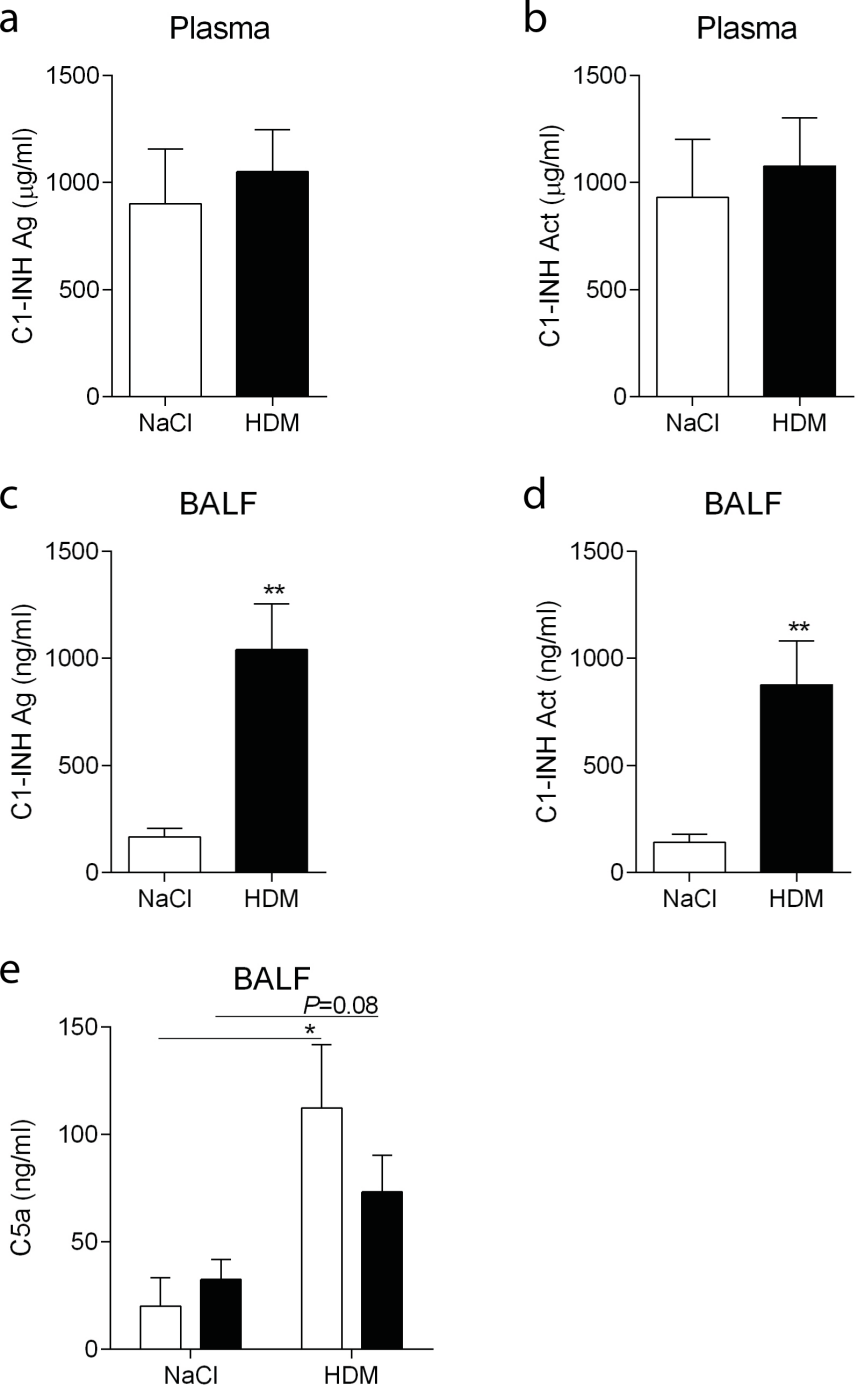
C1-INH and C5a levels in plasma and BALF. Human plasma derived C1-INH antigen (Ag) and activity (Act) in plasma (A-B) and BALF (C-D) of NaCl (white bars) and HDM (black bars) exposed C1-INH treated mice. Complement activation was determined in BALF (E) by C5a ELISA in vehicle (white bars) and C1-INH (black bars) treated NaCl and HDM exposed mice. Data are presented as mean ± SEM, n = 6 (NaCl) and n = 10 (HDM). *P<0.05, **P<0.01.

During the challenge phase of the HDM asthma model, mice received daily 15 U C1-INH or vehicle i.p. One day after the last challenge, and hence C1-INH administration, exogenous C1-INH antigen and activity levels were determined by ELISAs specific for human C1-INH. Similar plasma levels of either C1-INH antigen (Fig 2A) or C1-INH activity (Fig 2B) were observed following NaCl or HDM exposure. Although much lower than in plasma, presence of C1-INH, both antigen and activity, was confirmed in the BALF of NaCl and HDM treated mice (Fig 2C and 2D). Interestingly, C1-INH levels were significantly higher in BALF from HDM exposed mice as compared with the NaCl group. Of note, C1-INH antigen or activity could not be detected in vehicle treated NaCl or HDM mice indicating that there was no cross reactivity with mouse C1-INH.

To determine whether exogenous C1-INH was effective, complement activation was detected by an ELISA specific for a neoepitope on C5a. In BALF we could detect significant activation (4.2 ± 1.5 fold increase in ctr HDM versus ctr NaCl) of the complement system in vehicle treated mice, and a trend (*P* = 0.08; 2.1 ± 0.5 fold increase in C1-INH HDM versus C1-INH NaCl) towards higher BALF C5a in C1-INH treated mice following HDM exposure (Fig 2E). Although BALF C5a was lower in HDM exposed C1-INH treated mice, as compared with vehicle treated mice, this was not significant most likely due to intragroup variation. In plasma we could not detect activation of the complement system following HDM exposure.

### C1-INH treatment does not alter eosinophil influx

**Fig 3:**
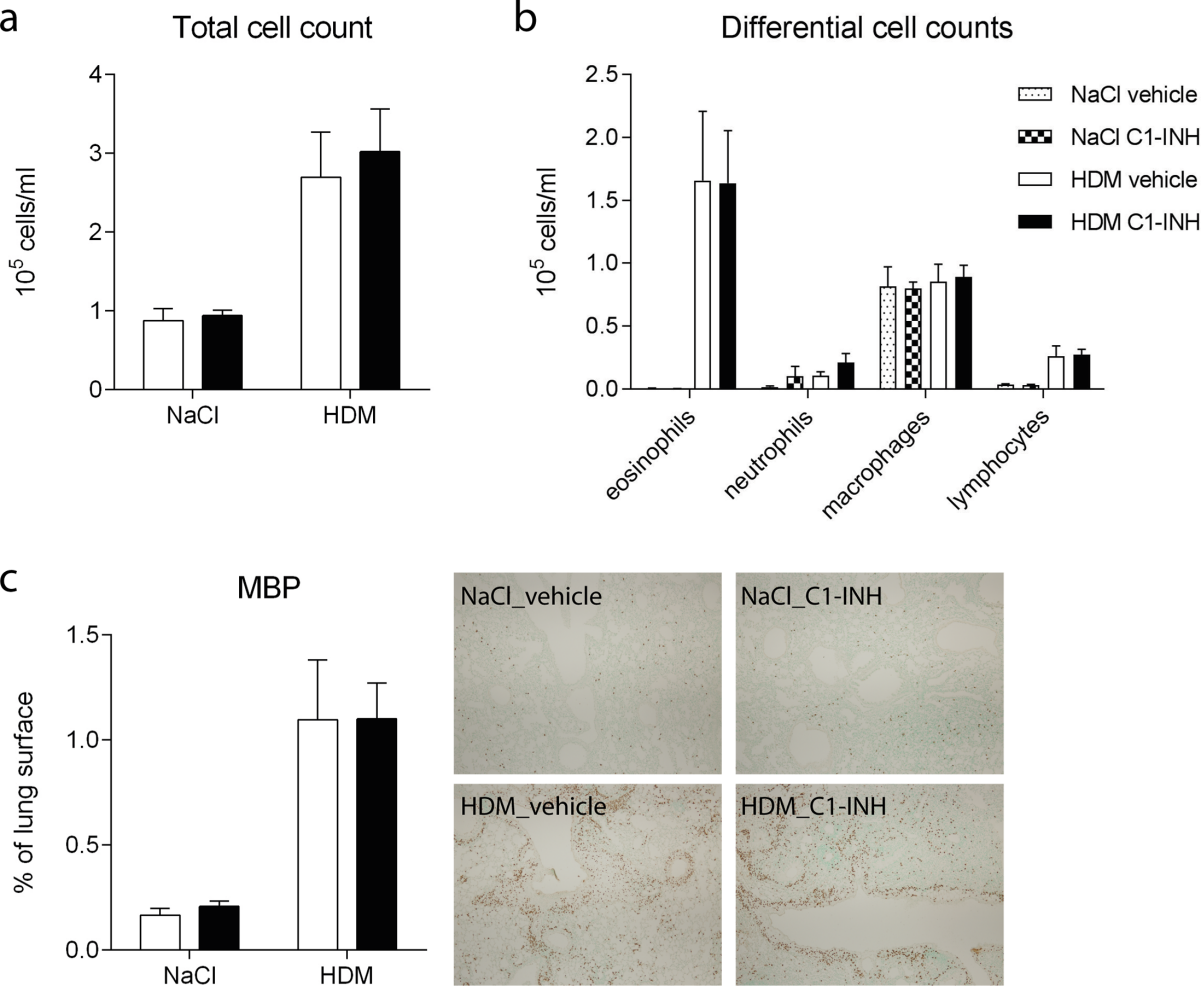
Eosinophil influx in BALF and lung. (A) Total cell count in BALF of NaCl or HDM challenged vehicle (white bars) or C1-INH (black bars) treated mice. (B) Differential cell count in BALF showing eosinophil, neutrophil, macrophage, and lymphocyte numbers in NaCl challenged vehicle (dotted bars) and C1-INH treated (blocked bars) mice and in HDM challenged vehicle (white bars) and C1-INH treated (black bars) mice. (C) Influx of eosinophils in the lung was determined by digital analysis of Major Basic Protein (MBP) stained lung sections in C1-INH (black bars) and vehicle (white bars) treated mice following challenge with NaCl or HDM. Representative pictures of each group are shown, magnification 4x. Data are presented as mean ± SEM, n = 6 (NaCl) and n = 10 (HDM).

One of the hallmarks of an asthmatic response is the influx of eosinophils in the lung. At time of euthanize the BALF was analyzed for total cell influx (Fig 3A) and the number of eosinophils, neutrophils, macrophages, and lymphocytes was assessed (Fig 3B). Following HDM exposure there was an approximately 3-fold increase in number of leukocytes, which was almost exclusively caused by eosinophils. C1-INH treatment had no effect on total leukocyte or leukocyte differential numbers in BALF of either NaCl or HDM exposed mice.

Additionally, eosinophil influx was determined in lung tissue by immunohistochemical staining for MBP, an eosinophil specific marker. In line with BALF influx data, more eosinophils were present in lungs of HDM exposed mice and no effect of C1-INH treatment was observed (Fig 3C).

### C1-INH treatment has no effect on lung pathology

**Fig 4:**
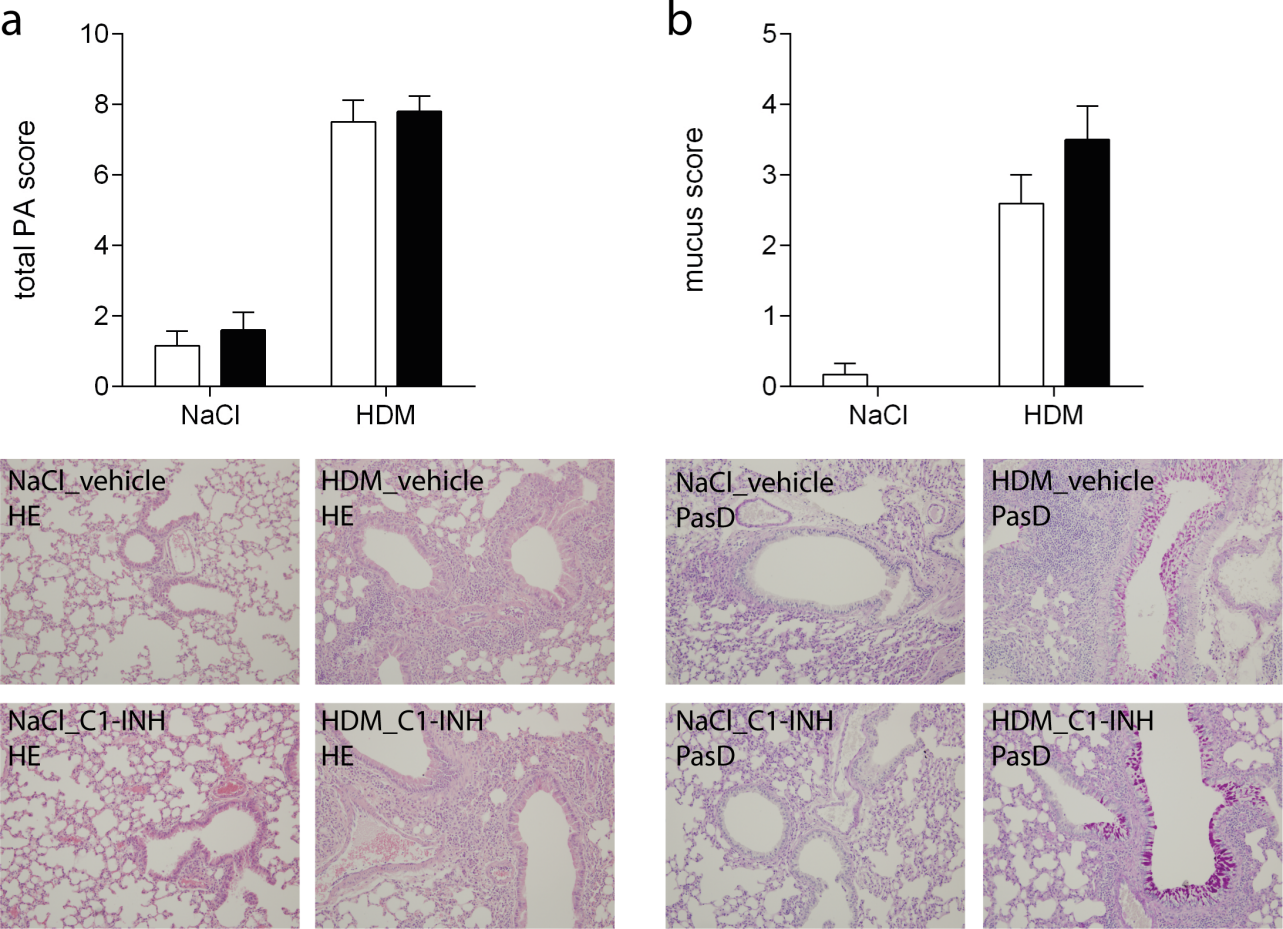
Lung damage and mucus production. Lung sections were stained with HE (A) or PasD (B) to determine damage, depicted as total pathology, or mucus production, respectively in NaCl or HDM exposed mice treated with C1-INH (black bars) or vehicle (white bars). Representative pictures of each staining per group are shown, magnification 10x. Data are presented as mean ± SEM, n = 6 (NaCl) and n = 10 (HDM).

Following HDM exposure histopathological changes, including perivascular inflammation, interstitial inflammation, and edema, occur in the lungs[16]. A pathologist, blinded for the treatment, analyzed these hallmarks in a semi-quantitative fashion and the total pathology score is depicted in Fig 4A. Although HDM exposure increased lung pathology, no effect of C1-INH treatment was observed.

One important feature of asthma is the increased production of mucus by bronchial epithelial cells[19]. Using a PasD staining to visualize mucus, lung sections were analyzed for the presence and localization of mucus resulting in a mucus score (Fig 4B). In line with total pathology, HDM exposure enhanced mucus production while C1-INH treatment had no effect hereon.

### C1-INH treatment inhibits Th_2_ response in MLN but not in BALF

**Fig 5:**
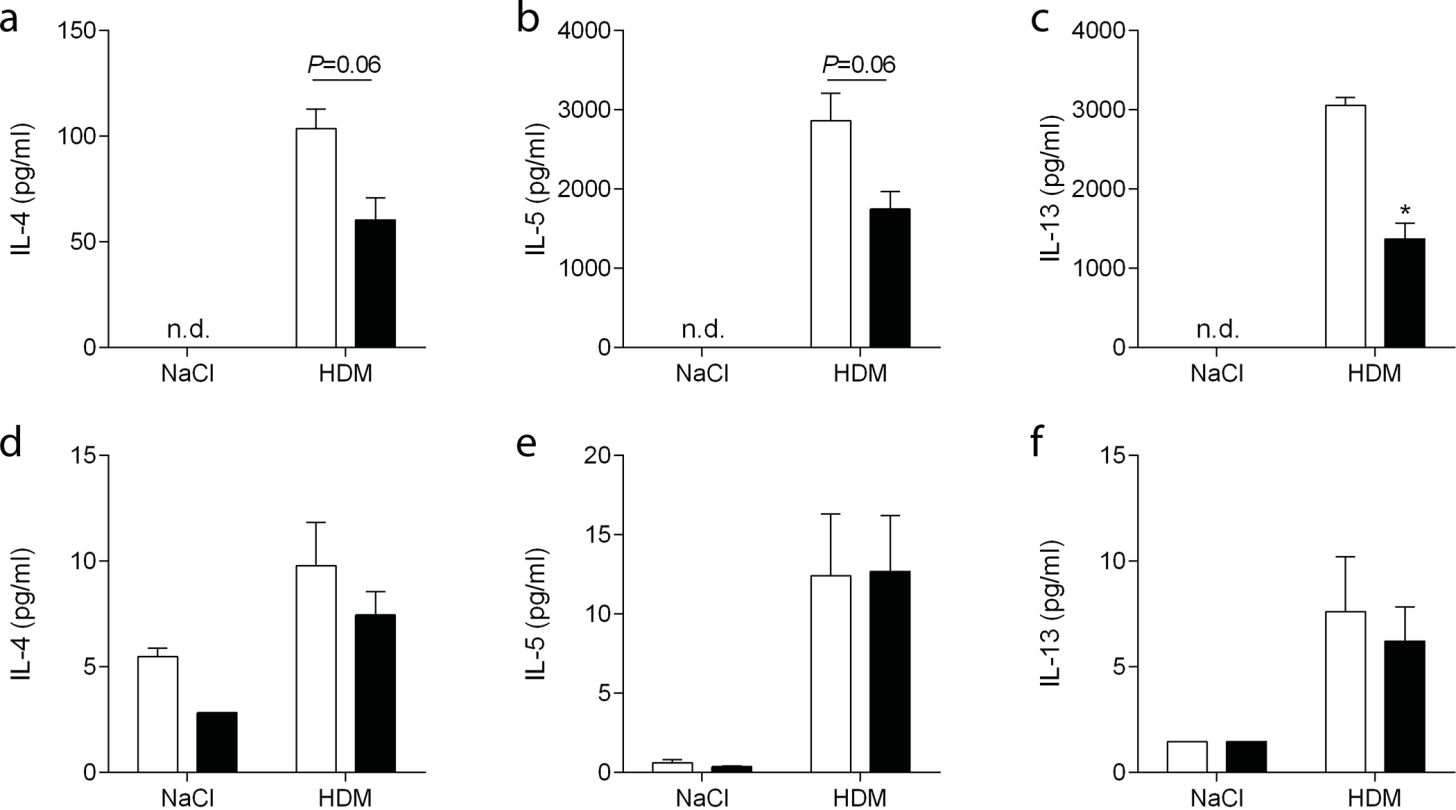
Th_2_ response in MLN and BALF. Following NaCl or HDM exposure MLN were dissected from mice treated with C1-INH (black bars) or vehicle (white bars). Equal numbers of MLN cells from C1-INH (black bars) or vehicle (white bars) treated HDM exposed mice were restimulated ex vivo with HDM and afterwards production of IL-4 (A), IL-5 (B), and IL-13 (C) was determined in the medium. In BALF of NaCl or HDM exposed mice treated with C1-INH (black bars) or vehicle (white bars) the cytokines IL-4 (D), IL-5 (E) and IL-13 (F) were determined by a magnetic luminex screening assay. Data are presented as mean ± SEM, n = 6 (NaCl) and n = 10 (HDM). *P<0.05.

In MLN, the draining lymph nodes of the lung, priming of T cells occurs[3]. To investigate the Th_2_ response following HDM challenge, we dissected MLN. Macroscopically we observed larger MLNs in HDM exposed mice as compared with NaCl groups. Interestingly, MLN in the C1-INH treated HDM group were smaller as compared with vehicle treated HDM mice. Equal numbers of MLN cells were seeded and restimulated with HDM or NaCl. IL-4, IL-5, and IL-13 secretion was measured as a determinant of the Th_2_ response. Only MLNs that were taken from mice challenged *in vivo* with HDM were used for this analysis. Following HDM restimulation MLNs from C1-INH treated mice had significantly lower IL-13 and a trend (P = 0.06) towards lower IL-4 and IL-5 production as compared with MLNs from vehicle treated mice (Fig 5A–5C). Incubation of MLN with NaCl did not induce a detectable cytokine response in either treatment group.

Next we evaluated Th_2_ cytokines IL-4, IL-5, and IL-13 in BALF. All three cytokine levels were low, however, increased following HDM exposure (Fig 5D–5F). C1-INH treatment did not significantly alter IL-13, IL-4, or IL-5 levels in the BALF.

### C1-INH treatment enhances HDM specific IgG_1_

**Fig 6:**
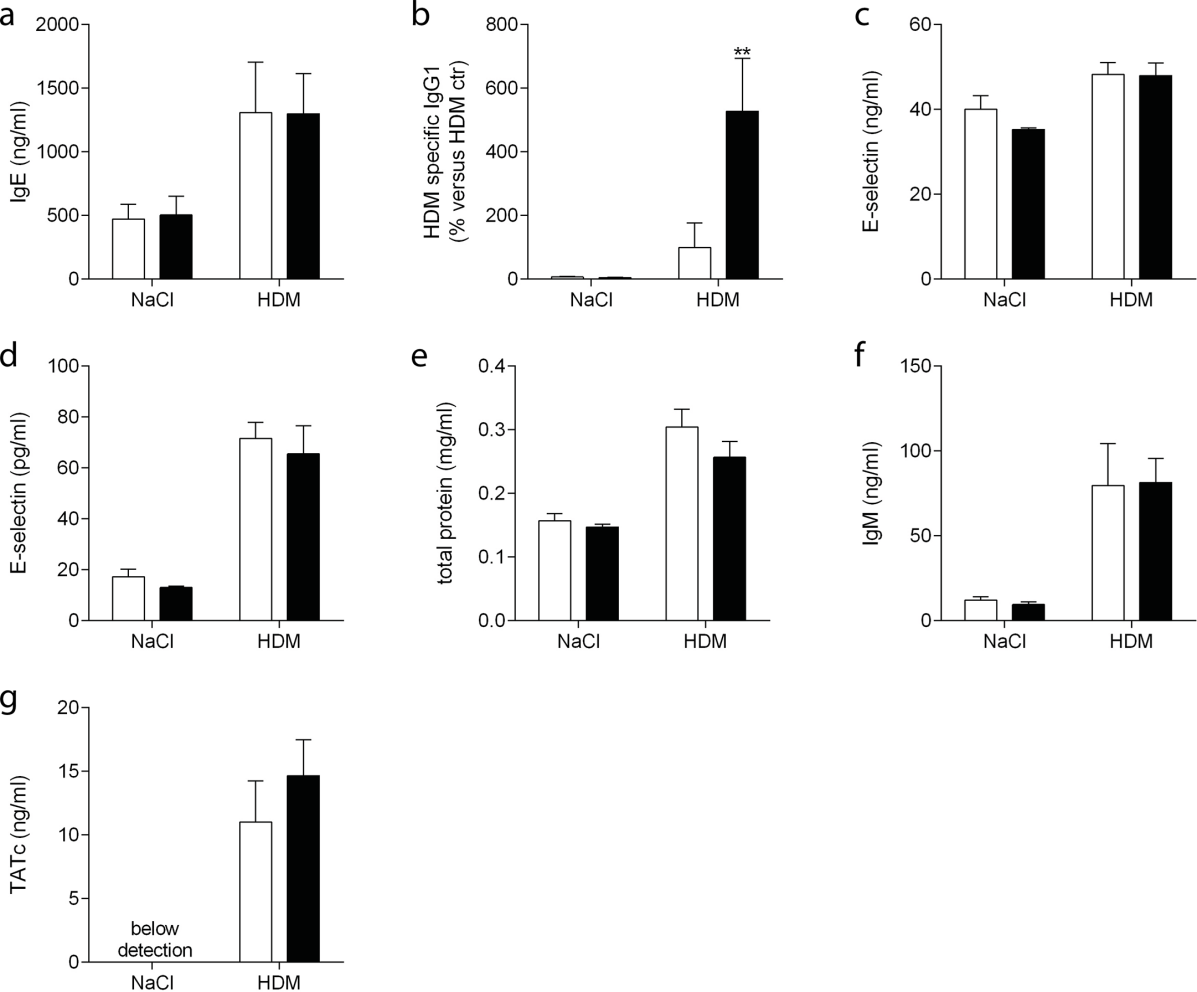
Plasma and BALF protein levels. In plasma of NaCl or HDM exposed mice treated with C1-INH (black bars) or vehicle (white bars) IgE (A), HDM specific IgG_1_ (B) and E-selectin (C) were determined. In BALF of C1-INH (black bars) or vehicle (white bars) treated NaCl or HDM exposed mice E-selectin (D), total protein (E), IgM (F) and TATc (G) were determined. Data are presented as mean ± SEM, n = 6 (NaCl) and n = 10 (HDM). **P<0.01.

Plasma IgE significantly increased following HDM exposure, however, C1-INH treatment did not affect this response (Fig 6A). To see whether the HDM specific antibody response was altered, we determined HDM specific IgG_1;_ there was a remarkably higher HDM specific plasma IgG_1_ response in C1-INH treated mice compared with vehicle treated mice.

HDM treatment induces vascular permeability[20] and C1-INH might affect this. We first measured plasma E-selectin as a marker of endothelial activation; C1-INH treatment had no effect on E-selectin plasma levels (Fig 6C). Additionally, we determined E-selectin, total protein and IgM in the BALF as markers of plasma leakage. Both increased following HDM exposure, however C1-INH treatment did not alter these markers (Fig 6D–6F).

C1-INH might reduce coagulation via inhibiting the coagulation proteins FXIa or FXIIa[7]. Therefore we analyzed TATc levels in BALF to determine activation of coagulation. HDM exposure induced TATc in the BALF of both vehicle and C1-INH treated mice (Fig 6G). C1-INH treatment had no effect on the extent of coagulation activation.

## Discussion

In the present study we determined the effect of human plasma-derived C1-INH in a clinically relevant HDM-induced asthma mouse model. C1-INH has already been proven to be beneficial in various inflammatory conditions. During lethal endotoxin shock, C1-INH protects against endothelial cell apoptosis, (micro)vascular permeability and mortality most likely via a direct effect on LPS that is independent of C1-INH protease inhibitor activity[21–23]. C1-INH protects against ischemia/reperfusion injury of the myocardium[8] and brain[24], early vein graft remodeling[25], and development of atherosclerosis[26]. Also in a TRALI (Transfusion Related Acute Lung Injury) model C1-INH has been proven to be effective in inhibiting complement activation and improving lung injury scores[27]. In our experimental setting we used a therapeutically relevant approach by administering C1-INH i.p. daily during the challenge phase, thereby circumventing a possible effect of C1-INH during the sensitization phase. It is for instance known that blocking C5a/C5aR signaling during the sensitization phase induces airway inflammation and hyperresponsiveness, while these parameters were reduced upon blocking of C5a/C5aR signaling during the challenge phase[28]. Although others have shown beneficial effects of C1-INH in inflammatory mouse models[8, 21–27], we did not observe a clinical effect of C1-INH treatment in our setting. In addition, recently, de Beer *et al*. reported no effect of C1-INH treatment in two different rat pneumonia models[29, 30].

Although several studies describing the effect of C1-INH in various experimental mouse models have been published, information about the pharmacokinetics of C1-INH in mice is limited. Therefore we analyzed the plasma C1-INH antigen and activity levels up to 72 hours after a single i.v. or i.p. injection. In humans the half-life of C1-INH is 28 hours[31], while the reported half-life in rats is 4½ hours[18]. To our knowledge, we are the first to describe the half-life of C1-INH in mouse, which is roughly 12 hours. Our aim was to reach plasma levels of 500–1000 μg/ml C1-INH in our experimental asthma model by administering once daily 15 U. At time of euthanize mice had C1-INH antigen plasma level of on average 900 μg/ml, 3 to 4-fold the human plasma concentration. C1-INH plasma levels were independent of HDM challenge. Human C1-INH antigen and activity BALF levels were elevated following HDM exposure. This is most likely due to an increase in plasma leakage, as confirmed by elevated BALF total protein and IgM. Human C1-INH antigen and activity levels were comparable in both plasma and BALF, indicating that all human C1-INH retained its protease inhibiting capacity. In line with previous studies in mice[16] and asthmatic patients[5], local activation of complement was seen in BALF. This was slightly inhibited by C1-INH treatment.

Following repeated exposure to HDM, mice developed a Th_2_ response as indicated by elevated levels of the cytokines IL-4, IL-5, and IL-13 in BALF and after restimulation of MLN. Following treatment with C1-INH, the Th2 cytokine response was lower in HDM restimulated MLN. Recently, a role for FXII in autoimmunity, independent of coagulation or contact-kinin system, was described[32]. In an experimental autoimmune encephalomyelitis mouse model, FXII deficiency or pharmacological blocking of FXIIa inhibited neuroinflammation and reduced influx of T cells in the brain[32]. The authors suggested that FXIIa acts on dendritic cells, thereby shaping T-cell differentiation and adaptive immunity[32]. As C1-INH is a potent inhibitor of FXIIa[7] and our results suggest a role for C1-INH in directing adaptive immunity, our data points into the same direction as the study by Göbel *et al*.[32]. Therefore, it would be of interest to see whether C1-INH would have therapeutic potential in autoimmune diseases such as multiple sclerosis.

Although the Th_2_ response was inhibited in MLN of C1-INH treated mice, systemic total IgE was comparable and HDM specific IgG_1_ was elevated following C1-INH treatment. From a classical point of view, a reduced Th_2_ response should go hand in hand with lowered (allergen-specific) antibody responses. However, allergen specific IgG competes with allergen specific IgE and thereby protects against allergic immune response, a mechanism that is used during allergen-specific immunotherapy[33]. Indeed, high-dose HDM exposure can induce IgG-mediated protection against HDM-specific anaphylaxis[34]. Therefore we hypothesize that in our HDM exposed asthma model elevated HDM specific IgG_1_ dampens adaptive immunity. Unfortunately we were not able to detect HDM specific IgE, as has been previously reported by others using the same HDM preparation[35, 36].

C1-INH is a major regulator of the contact-kinin system by blocking of activated FXII and plasma kallikrein, thereby suppressing formation of bradykinin. In experimental allergic airway models, several bradykinin receptor antagonists have been proven to be beneficial by, amongst other anti-inflammatory effects, reducing plasma leakage (reviewed in [12]). Moreover, in C1-INH deficient mice, human plasma-derived C1-INH reverses enhanced vascular permeability[37]. In the present study, however, we did not see an effect of C1-INH treatment on plasma leakage as similar IgM and total protein levels were detected in C1-INH and vehicle treated groups.

Several reports show an effect of C1-INH treatment on leukocyte chemotaxis[8–10]. This anti-inflammatory effect of C1-INH is presumably independent of its protease inhibitory activity and therefore cannot be ascribed to an indirect effect of complement pathway inhibition. In the present study, however, we did not observe an effect of C1-INH treatment on influx of eosinophils.

C1-INH is an inhibitor of fibrinolysis and may therefore lead to increased risk of thromboembolic complications. Indeed, several reports suggest an association between C1-INH use and risk of thrombosis[38], especially beyond the approved clinical indications and doses. However, the effect of high dose of C1-INH on thrombotic events is under debate. Recently, Schürmann *et al*. investigated the prothrombotic potential in rabbits and found no evidence for thrombosis at doses up to 800 IU/kg, but even a slight protective (anti-coagulant) effect of C1-INH in the FeCl_3_-induced arterial thrombosis model[39]. In line with the study from Schürmann, we did not observe a difference in coagulation activation using approximately the same dose of C1-INH. The observed adverse prothrombotic effects in clinical studies may be attributed to other causes such as the use of catheters or underlying thromboembolic risk factors, and further research is needed to confirm an association between thrombosis and C1-INH use[38, 40].

In the mouse C1-INH is highly expressed in the liver, but also relatively high in the heart and lung[41]. Moreover, airway epithelial cells induce expression of C1-INH in dendritic cells[42]. It can be speculated that the mouse lung contains high levels of C1-INH, which might even be increased following allergen exposure, to exert its function in the HDM asthma model. Hence human C1-INH treatment might result in excess C1-INH, and it is therefore no longer possible to delineate the effects of endogenous and exogenous C1-INH. A limitation of this study is the lack of endogenous C1-INH data in plasma and BALF. As far as we know, no data is available on the levels of C1-INH in asthmatic patients or experimental asthma models. It would be of interest to study the effect of C1-INH in mice that lack endogenous C1-INH.

In conclusion, C1-INH dampens the adaptive immune response by inhibiting Th_2_-induced cytokine production in MLN and by enhancing HDM-specific IgG_1_. These effects are most likely not mediated via blocking the complement, contact-kinin, or coagulation systems. Recent evidence suggests that C1-INH via blocking FXII might directly shape the adaptive immune response.
